# The effectiveness of cumulative interventions on the classroom management-related performance of pre-service teachers in physical education: improvement of knowledge and situation-specific skills alone is not sufficient

**DOI:** 10.1007/s11618-025-01358-4

**Published:** 2025-11-10

**Authors:** Matthias Baumgartner, Clemens Berthold, Eric Jeisy

**Affiliations:** Institute of Physical Education, Sport and Health (IPESH), St. Gallen University of Teacher Education, Seminarstrasse 27, 9400 Rorschach, Switzerland

**Keywords:** Practice-Based Teacher Education, Physical Education, Classroom Management, Professional Development, Performance, Intervention Study, Praxisorientierte Lehrer*innenbildung, Sportunterricht, Klassenführung, Professionelle Entwicklung, Performanz, Interventionsstudie

## Abstract

This intervention study, based on the competence model developed by Blömeke et al. (2015) and using classroom management (CM) in physical education (PE) as an example, investigates the effects of three developmental components. Specifically, it investigates how the following components influence the CM-related performance development of pre-service teachers in primary schools (PSTs): (1) improvement of CM-related knowledge, (2) enhancement of CM-related perception, interpretation, and decision-making skills (PID), and (3) implementation of CM-related quality criteria within one’s own teaching practice. Four interventions were carried out in which all three CM-related competence facets (knowledge, PID, and performance) were measured before and after the respective interventions. One intervention group (*IG*_*1*_) participated solely in an internship. Targeted interventions were developed for IGs_2–4_, creating cumulative interventions, whereby a further development component was integrated into the next higher intervention stage in addition to the internship. The results indicate that a general internship (*IG*_*1*_) had no impact on the performance development of the PSTs. In IG_2_, targeted interventions led to a significant advancement in knowledge, and in IG_3_, both knowledge and PID improved significantly, but this had no influence on the performance development. Only in IG_4_ was a significant progression of performance observed. The results indicate that improvements in CM-related knowledge and CM-related PID alone do not lead to an enhancement in CM-related performance. Instead, progress in CM-related performance is achieved through teaching and learning arrangements that incorporate additionally the components of coaching, video-based feedback, self-reflection, and training.

## Abstract (de)

In der vorliegenden Interventionsstudie, die sich am Kompetenzmodell nach Blömeke et al. (2015) orientiert und das Beispiel der Klassenführung (KF) im Sportunterricht heranzieht, werden die Effekte von drei Entwicklungskomponenten auf die KF-bezogene Performanzentwicklung bei angehenden Lehrkräften der Primarschule untersucht. Diese Komponenten umfassen (1) die Verbesserung des KF-bezogenen Wissens, (2) die Verbesserung der KF-bezogenen Wahrnehmungs‑, Interpretations- und Entscheidungsfähigkeiten (WIE) sowie (3) das Üben der Umsetzung der KF-bezogenen Qualitätskriterien in der eigenen Unterrichtspraxis. Dabei wurden vier Interventionen durchgeführt, in welchen vor und nach den Interventionen jeweils alle drei KF-bezogenen Facetten (Wissen, WIE, Performanz) gemessen wurden. Eine Untersuchungsgruppe (*UG*_*1*_) absolvierte einzig ein Schulpraktikum. Für UG_2–4_ wurden Interventionen entwickelt, wobei zusätzlich zum Praktikum jeweils eine weitere Entwicklungskomponente in die nächste höhere Stufe integriert wurde. Die Ergebnisse zeigen, dass das Praktikum (*UG*_*1*_) keinen Einfluss auf die Performanzentwicklung der Untersuchungsteilnehmenden (UT) hatte und sich durch die gezielten Interventionen in UG_2_ das Wissen und in UG_3_ das Wissen und die WIE signifikant verbessert haben, jedoch nicht die Performanz. Einzig in IG_4_ zeigt sich ein signifikanter Performanzfortschritt. Die Resultate zeigen auf, dass die Verbesserung des KF-bezogenen Wissens und der KF-bezogenen WIE allein nicht zu einer Verbesserung der KF-bezogenen Performanz führt. Der KF-bezogene Performanzfortschritt erfolgte in einem Lehr- und Lernarrangement, in welchen zusätzlich die Komponenten Coaching, videobasiertes Feedback, Selbstreflexion und Training berücksichtig wurden.

## Introduction and research question

**Fig. 1:**
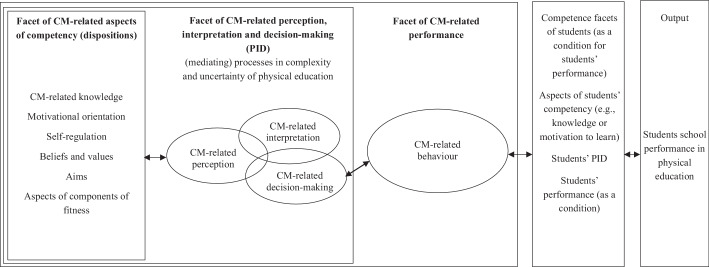
CM-related professional competence of physical education teachers (adapted from Baumgartner 2018a, 2022a)

In studies on the effectiveness of teacher education and professional development, researchers often focus on (developing) particular aspects of competency (e.g., professional knowledge) or situation-specific skills (e.g., perception). However, this approach neglects the application of these aspects of competency or skills in the complex, real-world teaching context, which demands additional effort from (pre-service) teachers (PSTs; cf. Baumgartner 2022a). Recently, there has been a shift in teacher education (research) towards a more performance-oriented approach (e.g., Albu and Lindmeier 2023; Brataas and Jenset 2023). This—in our view—more realistic approach emphasizes the evaluation of teachers based on their performance in real-world settings (cf. Baumgartner 2018a). The tendency towards a stronger focus on performance is also evident in the model of “competence as a continuum” (Blömeke et al. 2015, p. 11), in which performance has been integrated into the competence construct. This model has been adapted and extended for the professional competences of physical education (PE) teachers (Baumgartner 2017, 2022a). The competence framework draws upon three competence facets: 1) aspects of competency (dispositions, e.g., professional knowledge), 2) the situation-specific skills of perception, interpretation, and decision-making (PID), and 3) performance (cf. Baumgartner et al. 2023).^1^ Moreover, we interpret these three facets as related to competence areas (e.g., classroom management [CM], feedback, etc.), aligning with the holistic, competence-oriented tradition (e.g., Oser 2013; cf. Baumgartner 2022a). When performance is considered the target variable, the aspects of competency and PID rigorously serve the facet of performance. In turn, the facet of performance rigorously serves the (positive) development of students (see Fig. 1).

The model (cf. Baumgartner 2022a) is grounded in a developmental assumption that is based on three components of development that build cumulatively upon one another: the first component involves improving aspects of competency, such as improving professional knowledge through literature reviews. The second component centres on enhancing PID, for example, by analysing classroom videos (of others). The third component focuses on the practice of applying the quality criteria—such as those for effective CM—in one’s own teaching practice (e.g., Ericsson et al. 1993; Wilkinson et al. 2020; cf. Baumgartner 2018b). This third component involves structured opportunities to apply these criteria in practice, integrating elements such as coaching, (video-based) performance feedback, training, and self-reflection (see Wilkinson et al. 2020 and Fig. 1).

At present, no (quasi-)experimental studies are available that have empirically examined these three facets of competence in an area-related specification (e.g., CM-related knowledge, CM-related PID, and CM-related performance) or assessed the influence of these three components of development on the improvement of performance. Using the example of CM among pre-service physical education (PE) teachers, this research gap is addressed by the project “From Knowledge to Performance in Physical Education: Pre-Service PE Teachers’ Transformation of Competences—An Intervention Study on Classroom Management (WiPe-Sport)”, supported by the Swiss National Science Foundation (SNSF). In an intervention study, the project will investigate to what extent the quality of the CM-related performance of pre-service teachers (PSTs) in PE can be improved through the three components of development.

## Theoretical framework

### Classroom management (in PE)

CM is a key competence area for (PE) teachers and a critical factor in effective teaching (Baumgartner et al. 2020; Brophy 2006; Herrmann and Gerlach 2020; Weyers et al. 2024). Research links high-quality CM to students’ academic outcomes, attention, engagement, and motivation (Brophy 2006; Korpershoek et al. 2016). For (novice) teachers, effective CM is also associated with reduced stress (Dicke et al. 2015).

In PE, CM refers to “the sum of teachers’ actions that aim to create a conducive learning environment for cognitive, social-emotional, and motor learning” (Baumgartner et al. 2020). CM in PE involves specific challenges: diverse teaching environments (e.g., gyms, outdoors), high noise levels, frequent transitions, and safety concerns due to physical and psychological vulnerability (Baumgartner et al. 2020; Cothran and Kulinna 2015). Consequently, the importance of monitoring (e.g., oversight in large halls) and managing smooth transitions (e.g., handling equipment) is particularly high and differs qualitatively from other subjects. Ineffective CM is linked to reduced student motivation (Miethling and Krieger 2004), while effective CM is associated with increased motivation (Heemsoth 2014). PE teachers consistently report CM as both essential and demanding (Baumgartner 2013).

We conceptualize CM closely in line with the competence model as developed by Blömeke et al. (2015), specifically adaption and extension according to Baumgartner (2018a, 2022a). Accordingly, the CM-related competences of PE teachers are constituted by CM-related aspects of competency (in this study knowledge), CM-related PID, and CM-related performance. In turn, the facet of CM-related performance rigorously serves the development of students (cf. Fig. 1).^2^

### Does the improvement of (CM-related) professional knowledge lead to higher (CM-related) performance?

Professional knowledge is a crucial aspect of teacher competency and often outweighs factors like motivational orientation or beliefs (Baumert and Kunter 2006; Guerriero 2017; Kaiser and König 2019). CM-related knowledge, embedded within general pedagogical knowledge (GPK), supports teachers in creating effective learning environments across subjects (Guerriero 2017). Although GPK is positively associated with teaching quality and student outcomes (Ulferts 2019), the generalizability of these findings is limited by the variability in GPK assessment methods, including differences in content dimensions, subject focus, contextualization, and data collection (Brühwiler and Hollenstein 2021)—which can lead to contradictory results (Brühwiler et al. 2017). Therefore, it is necessary to specify these aspects when establishing connections to other competence facets.

Studies focusing specifically on CM-related performance as a distinct aspect of teaching quality report small to moderate associations with GPK, based on both student perceptions and classroom observations (König et al. 2021; König and Pflanzl 2016; Lenske et al. 2016). Lenske et al. (2016) demonstrated that GPK exerts both direct effects on students’ content knowledge and indirect effects mediated by observed teacher classroom-management performance. However, broad GPK assessments may overlook CM-specific knowledge (Römer and Rothland 2015), and its effects may diminish when considered separately from the broader GPK construct (e.g., Junker et al. 2021). Furthermore, the predictive power of CM-related knowledge depends on effects regarding the type of knowledge being tested. Conditional-procedural CM knowledge, which is application-oriented, has been shown to be a more effective predictor of performance than declarative knowledge (Lenske et al. 2016). Yet, conditional-procedural knowledge may overlap with measures of PID or performance, as it involves, for example, evaluating responses to classroom scenarios (Lenske et al. 2016) or is inferred from written reflections (Weber et al. 2023).

In conclusion, CM-related knowledge generally appears to positively impact CM-related performance, but findings remain inconsistent and contingent upon the specific aspects of CM-related knowledge being measured.

### Does the improvement of (CM-related) PID lead to higher (CM-related) performance?

Effective CM requires teachers to perceive CM-relevant events, interpret them based on their knowledge, and make situation-specific decisions to support student learning. These skills, referred to here as CM-related PID (following Blömeke et al. 2015), are also known as *professional vision* (Gold and Holodynski 2017) or *noticing* (König et al. 2022). While these concepts broadly address situation-specific skills, their theoretical frameworks differ (König et al. 2022). This study conceptualizes CM-related PID as knowledge-based cognitive demands linking knowledge to performance, aligning with Blömeke et al. (2015; see Fig. 1). Empirically, PID is assessed through diverse methods, ranging from analytical or holistic testing methods to data collection approaches *on action* (e.g., video-based testing instruments) and *in action* (e.g., eye-tracking studies; see Weyers et al. 2023).

Studies along the knowledge-to-performance continuum suggest a link between CM-related knowledge and CM-related PID (e.g., Gippert et al. 2022; Gold and Holodynski 2017). The relationship between CM-related PID and performance is less clear. König and Kramer (2016) found a moderate effect at the secondary school level, showing that professional perception of CM is a valid predictor of students’ ratings of the quality of their teacher’s CM. However, Gold et al. (2021) did not find a significant relationship between professional vision and the student-rated CM performance of teachers (primary school). Similarly, Junker et al. (2021) did not find any significant correlations between professional vision and observed CM-related performance among beginner in-service teachers. High performance scores and low PID levels in these studies may have limited variance. Model-based analyses (Blömeke et al. 2022) support the theoretical link from knowledge to performance via PID, but lack causal confirmation due to cross-sectional designs.

In summary, PID appears to support CM performance, but findings remain mixed and depend on conceptual and methodological factors.

### Does the practice of applying the CM-related quality criteria in one’s own teaching practice lead to higher performance?

An additional challenge of good teaching is the situational complexity (e.g., Baumgartner 2017; Doyle 2006). According to Doyle (2006), (PE) teaching is multidimensional, requiring teachers to respond immediately and in a goal-oriented manner to situations. Even with detailed planning, teaching scenarios remain often unpredictable. This situational complexity of (PE) teaching is one reason why (PE) teachers cannot automatically apply their professional knowledge or PID in their practice. When examining the existing evidence on the effectiveness of interventions to improve the CM-related performance of (pre-service) teachers, the results are inconsistent (cf. Wilkinson et al. 2020). One reason for this inconsistency is the variety of intervention designs, which incorporate different components (e.g., microteaching sequences, analysis of one’s own vs others’ classroom videos), and the variety of intensity and duration (cf. Brouwer 2022; Wilkinson et al. 2020).

In their review of the literature on the improvement of CM-related performance, Wilkinson et al. (2020) emphasize that effective interventions typically include components such as coaching, video-based performance feedback, structured self-reflection, and training. Studies like Piwowar et al. (2016) and Hickey et al. (2017) support this view: interventions combining knowledge with video analysis, and performance feedback led to moderate to large improvements in observed CM performance. In contrast, general internships without structured support showed no effect (Bach 2013; Baumgartner 2018b; Greve et al. 2020).

In summary, effective interventions on the improvement of CM-related performance incorporate various components with coaching, video-based performance feedback, self-reflection, and training being particularly effective (Wilkinson et al. 2020).

## Objective, research question, and hypotheses of the present study

The objective of the present study is to generate evidence regarding the effects of the components that build upon each other: 1) enhancement of CM-related knowledge, 2) improvement of CM-related PID, and 3) the practice of applying the quality criteria of CM in one’s own teaching practice on the development of CM-related performance among PST of PE.

Guided by the competence model (Baumgartner 2022a), the study follows a cumulative developmental logic. It examines whether CM-related performance among PST of PE improves with the successive addition of targeted developmental components. Based on prior research, no significant change is expected from a general internship alone. A small improvement is anticipated when CM-related knowledge is added. A moderate improvement is expected when PID are addressed in combination with knowledge. A strong improvement is expected when all three components—including the practice of applying the quality criteria (of CM) in one’s own teaching practice—are implemented (see 2.2–2.4). The effects of these components are investigated across four intervention stages, leading to the following hypotheses:

### H_1_

The quality of the CM-related performance (dependent variable, *DV*) of PSTs who complete an internship (independent variable, *IV*_*1*_) does not change significantly.

### H_2_

The quality of the CM-related performance (*DV*) of PSTs who complete an intervention programme (*IV*_*2*_) to enhance their CM-related knowledge as well as an internship improves significantly (small effect).

### H_3_

The quality of the CM-related performance (*DV*) of PSTs who complete an intervention programme (*IV*_*3*_) to enhance their CM-related knowledge and PID as well as an internship improves significantly (moderate effect).

### H_4_

The quality of the CM-related performance (*DV*) of PSTs who complete an intervention programme (*IV*_*4*_) to enhance their CM-related knowledge and PID, as well as the practice of applying the quality criteria of CM in one’s own teaching practice, improves significantly (strong effect).

## Methods

### Study design and variables

A quasi-experimental field study was conducted with four intervention groups (*IG*_*1*_*–IG*_*4*_), each receiving a different combination of developmental components. The main outcome variable was the quality of CM-related performance at post-test (*t*_*2*_), while the corresponding pre-test score (*t*_*1*_) was included as a covariate. Similarly, the improvement in both CM-related knowledge (*H*_*2–4*_) and PID (*H*_*3–4*_, cf. 4.3) were assessed, to check whether all competence facets were adequately addressed in the interventions. Both constructs were additionally used as control variables (*CVs*) to isolate the effects of the different developmental components on CM-related performance. The type of internship (general mandatory full-time vs voluntary specialized PE; see 4.3), age and gender were included to account for environmental and sample influences.

### Sample

The study involved 80 PSTs from the St. Gallen University of Teacher Education (PHSG), specializing in primary education, enrolled in either a general mandatory internship or a voluntary specialized PE internship. Over three years, the PSTs study 10 subjects (including PE). The intervention participants (*IPs*) were selected based on their enrolment in either a general mandatory full-time internship or a voluntary specialized PE internship and were invited to take part in the research. In this regard, IG_1_ and IG_2_ comprised two pre-existing learning groups in their sixth semester at PHSG, randomly selected from a total of 10 groups. These IPs were placed in single or mixed grade classrooms across 28 different schools in the region St. Gallen as an integral component of their standard teacher education programme. IG_1_ included 19 IPs (18 = *f.*, 1 = *m.*, 0 = *d.*) with an average age of 22.7 years (*SD* = 2.18, *range* = 21–30), while IG_2_ encompassed 19 IPs (19 = *f.*, 0 = *m.*, 0 = *d.*) with an average age of 23.7 years (*SD* = 2.85, *range* = 21–31). IG_3_ and IG_4_ consisted of two learning groups of PSTs in their fourth or sixth semester that had voluntarily enrolled in a specialized PE internship teaching mixed grade groups. This internship was selected as it provides a more focused and structured experience in PE, allowing for a higher dosage of intervention (see 4.3). IG_3_ comprised 20 IPs (11 = *f.*, 9 = *m.*, 0 = *d.*), with an average age of 22.8 years (*SD* = 2.02, *range* = 20–26). IG_4_ consisted of 22 IPs (11 = *f.*, 11 = *m.*, 0 = *d.*), with an average age of 23.6 years (*SD* = 4.73, *range* = 20–40). Of the IPs, 17 taught students in grades 1 and 2, 15 taught students in grades 3 and 4, and 10 taught students in grades 5 and 6.

### Interventions

**Fig. 2:**
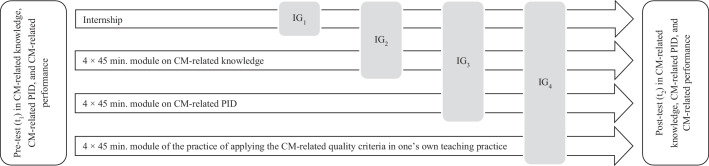
The cumulative structure of the interventions, designed to integrate each developmental component respectively

Four IGs received increasingly comprehensive interventions based on a cumulative design. Beyond the internship, each successive intervention stage incorporated an additional development component (see Fig. 2). Throughout the study, the same researcher delivered standardized intervention modules to each group.

#### IG_1_

IG_1_ (standard intervention) participated in a general mandatory full-time teaching internship, during which IPs were assigned a class and taught 80%–100% of all their lessons across all subjects. These encompass three PE lessons per week, throughout the investigation (approx. three weeks). The IPs were supervised by the classroom teacher and monitored by a university mentor. However, the IPs did not receive specific feedback on their CM.

#### IG_2_

IG_2_ also participated in the same internship as IG_1_. Additionally, IG_2_ took part in a module designed to improve their CM-related knowledge (4 × 45 min.), conducted by the research team. This intervention consisted of three sessions aimed at improving the IPs’ CM knowledge for PE. In the first session (2 × 45 min.), the IPs were introduced to the nine-dimensional framework of effective CM in PE, as outlined by Baumgartner et al. (2020). This session involved discussing text-based case studies that aligned with or deviated from this framework, with a focus on an action-oriented understanding of CM. In the second session (1 × 45 min.), the IPs worked through specific classroom scenarios designed to broaden their repertoire of evidence-based CM strategies across various CM dimensions. In the final session (1 × 45 min.), the IPs were encouraged to apply their newly acquired knowledge by creating a teaching script for an upcoming PE lesson. This required them to develop action plans to anticipate and prevent potential CM challenges in their upcoming lesson. The intervention in IG_2_ involved only text-based activities. To avoid directly targeting the PID, the use of videos was omitted here.

#### IG_3_

IG_3_ participated in a voluntary specialized PE internship (lasting five days) during the school holidays. The participating primary school students attended four consecutive PE lessons each day. In groups of four, the IPs were assigned a group of students to supervise and teach throughout the internship. Each IP was required to teach one PE lesson per day. IG_3_ undertook the module to develop CM-related knowledge and attended a second module to enhance CM-related PID (4 × 45 min.). In the first session (2 × 45 min.), the IPs were introduced to key concepts and the current state of PID research. They discussed CM-related video vignettes of other teachers’ teaching developed for the test instrument (Baumgartner et al. 2023; Jeisy et al. in press), which were not included in the final version. In the second session (1 × 45 min), the IPs practised their PID utilizing the CM section on the *bewegunglesen.ch* platform (Baumgartner et al. 2021). During the third session (1 × 45 min.), small groups of IPs developed their own video vignettes based on a 10-minute clip of other teachers’ PE lessons. They accomplished this by tagging specific time frames in the video and then using the CM observation instrument to formulate items for one self-selected CM dimension. Pairs of groups then presented their vignettes and items to each other for detailed content discussion. To avoid directly targeting their own performance, video vignettes from other teachers were used in this intervention. Interventions on CM-related knowledge and PID were delivered in an interwoven structure across the week, starting on Monday with the first knowledge session, progressing on Tuesday to both the second knowledge session and the first PID session, alternating on Wednesday with one of each, and concluding on Thursday with the final PID session.

#### IG_4_

IG_4_ undertook the same five-day internship and completed the same two modules as IG_3_, following the identical sequence of delivery. Additionally, IG_4_ participated in a third module incorporating instructor coaching, video-based performance feedback, self-reflection, and training. All five of the IPs’ lessons were videotaped. Following each daily lesson, the IPs received the video of their lesson for video- and portfolio-based reflection involving the observation instrument to tag relevant CM-related scenes via the tool *frame.io*. This self-reflection process lasted approximately 45 min. per day. An instructor reviewed these reflections, provided written performance feedback on the tags, and added further annotations. Finally, the IPs and their instructor discussed the videotaped lesson and tags, and developed specific actions to improve their CM for the upcoming lesson, which followed the next day. This performance feedback process lasted approximately 30 min. This process (self-reflection and performance feedback) was repeated over four days.

### Instruments, measurement and data collection

The quality of CM-related knowledge, PID, and performance was assessed before (*t*_*1*_) and after (*t*_*2*_) the interventions. For IG_1_ and IG_2_, the first dataset (*t*_*1*_) was collected one week after the start of the internship, and the second set (*t*_*2*_) three weeks later, in March 2023. For IG_3_ (April 2023) and IG_4_ (October 2022), data were gathered on the first (*t*_*1*_) and fifth (*t*_*2*_) days of the internship week.

#### CM-related performance (*DV*)

In the present study, we adopt a broad understanding of CM-related concepts to ensure high ecological validity. Accordingly, we used a comprehensive observation tool to measure the CM-related performances of PE teachers, which includes both general dimensions of good CM (e.g., monitoring) and subject-specific dimensions (e.g., safety; Baumgartner et al. 2020). The instrument evaluates nine quality dimensions (latent variables) using 27 items (manifest variables). Each item is rated on a four-point Likert scale (1 = does not apply, 2 = rather does not apply, 3 = rather applies, 4 = applies), with an additional option of “cannot be assessed” (NA; see Table 1). The quality of CM-related performance was determined by calculating the mean score across all items.

**Table 1:** Instrument for measuring CM-related performances in PE teaching (Baumgartner et al. 2020)

Dimensions	Items (*n*)	Example of item. The PE teacher …
Monitoring	4	… chooses positions in the room from which she/he has a good overview of what is going on in the class
Safety	3	… organizes the learning environment safely (e.g., use of gym mats)
Clarity of announcements	3	… formulates objectives/tasks/exercises in a comprehensible way
Dealing with disruptions	3	… manages to focus the learners’ attention on the lesson in the case of minor disturbances
Use of material	3	… does not use too much equipment and materials in PE lessons
Momentum	3	… progresses quickly in terms of content so that there is no waste of time
Smooth transitions	3	… intervenes in a targeted way when transitions do not run smoothly
Overlapping	3	… can dedicate himself/herself to a teaching event without neglecting the class
Group mobilization	2	… enables many learners to learn at the same time

At both t_1_ and t_2_, full PE lessons (approx. 45 min.) were taped from two camera perspectives (a fixed classroom camera and a mobile interaction camera) and the verbal interactions between the IPs and students were recorded using a wireless microphone. At both t_1_ and t_2_, the IPs instructed the same primary school classes in the same content area (e.g., athletics).^3^

To assess the CM-related performance of the IPs, a rating process was conducted. The video rating process took place between May and August 2023. Pre-service secondary school teachers at PHSG, specializing in PE, were invited to serve as raters due to their advanced knowledge in the field. Faculty members selected suitable candidates, who were then offered paid research assistant positions. Five raters (*M* = 23.4 years, *SD* = 2.6, 4 = *f.*, 1 = *m.*, 0 = *d.*) from the sixth and eighth semesters participated after completing a training programme to ensure consistency and reliability in their assessments. To ensure a high inter-rater reliability (*ICC*), the five raters underwent a structured four-stage training programme.

In the first stage, raters undertook self-study to familiarise themselves with CM in PE and the observation tool (Baumgartner et al. 2020). In the second stage, the project team introduced the rating process in a workshop, addressed raters’ questions, and facilitated the evaluation of a practice teaching session. In the third stage, raters worked in pairs to assess additional practice videos, then compared and reconciled their assessments with the project team. Finally, each rater independently assessed further practice videos and, where necessary, discussed their judgments within their own team. After the training, the raters assessed the IPs’ CM-related video vignettes. Each IPs’ two videos (*t*_*1*_, *t*_*2*_) were randomly assigned to a rater, with the raters advised to rate both videos of the same IP consecutively. To prevent expectation effects, the raters assessed the videos without knowing which time point they were evaluating. To ensure consistency, the raters convened after 10 videos to discuss a training lesson and resolve discrepancies. Each rater assessed 42–44 videos (approx. 45 min. each), with 50% of the lessons being double scored. Overall, a total of 146 videos were scored (220 incl. double scoring).

#### CM-related knowledge (*CV*) and situation-specific CM-related PID (*CV*)

CM-related knowledge was measured utilizing a self-developed test instrument aligned with the observation tool (Baumgartner et al. 2020; Berthold et al. 2025). This test draws on evidence related to the nine dimensions of CM depicted in the observation instrument and assesses declarative, non-situated knowledge about effective CM practices. This approach aims to distinguish knowledge from PID, enabling their separate assessment, while closely aligning the content dimensions. Dichotomous items were validated qualitatively through a Delphi study (Baumgartner et al. 2023) and quantitatively using a sample (*n* = 877) of PSTs (Berthold et al. 2025). Psychometric analysis by Berthold et al. (2025) provided evidence of construct validity for a unidimensional structure under a two-parameter (*2PL*) item response theory (*IRT*) model exhibiting sufficient reliability (*EAP* = 0.603; *WLE* = 0.569). The computer-based test comprised 79 items, such as: “To monitor the classroom, a teacher should choose a fixed position to see all students” (false, dimension of monitoring).

The CM-related PID was measured with a self-developed, video-based test instrument aligned with the observation tool (Baumgartner et al. 2020). The PID test included short video vignettes (1:19–3:27 min.) that depicted all nine dimensions of CM at least twice. The video vignettes were used as item prompts. They were derived from video-recorded PE lessons created during previous specialized PE internships at PHSG. These lessons were conducted with same-aged children and students and cover the content areas of Gymnastics and Games. For each CM-related video vignette, dichotomous video-specific items were formulated to evaluate the three cognitive demands of PID separately. This test was validated through a Delphi study (Baumgartner et al. 2023) and a sample (*n* = 877) of PSTs (Jeisy et al. in press). Psychometric analyses conducted by Jeisy et al. (in press) supported a unidimensional structure under a 2PL-IRT model for the situation-specific skills, indicating that the instrument captures PID as a single construct. The reliability indices were sufficient (*EAP* = 0.674; *WLE* = 0.639). The test comprised seven CM-related video vignettes (total runtime: 15:15 min.) and 104 test items. An example item is: “Which aspect or aspects of CM did you perceive as particularly relevant in the teaching sequence shown? 1. Monitoring, 2. Clarity of instructions, 3. …”. The average combined test duration for knowledge and PID was 38 min. (*SD* = 11.0).

### Data analysis

Data quality for CM-related performance (interrater reliability) was evaluated with the intraclass correlation coefficient (*ICC [3, k]*), calculated separately for each rating team (one pair and one triplet). Missing data were addressed utilizing pairwise deletion, allowing us to include IPs with missing values in a single facet. Outliers, defined as data points exceeding three standard deviations from the mean, were reviewed. Data distributions were checked to ensure they met the assumptions required for statistical tests. Z‑standardized values of skewness and kurtosis exceeding ± 2.58 indicated a deviation from normality. The Shapiro-Wilk test and the Levene test were employed to assess the normality of residuals and homogeneity of variances between groups, respectively.

Data were centred at baseline (*t*_*1*_) to aid in the interpretation of analysis of covariance (*ANCOVA*) results, facilitating accurate comparisons over time. Data preparation was necessary to obtain person estimates from the IPs’ answers in the knowledge and PID tests. Since the test instruments were initially validated using one-dimensional two-parameter logistic item response theory (*2PL-IRT*) models, we estimated the person parameters based on the fixed item parameters from the previous validation studies (Berthold et al. 2025; Jeisy et al. in press). Consequently, a person estimate of 0 corresponds to the average ability level of IPs in the original validation study. At t_1_, we calculated EAP and WLE reliabilities for our sample to reflect the measurement precision of person-ability estimates produced by the IRT model. In general, reliability coefficients above 0.50 are considered acceptable for group comparisons, while those above 0.70 indicate a good test instrument (Wess et al. 2021).

The analysis aimed to uncover the impact of IG participation (*IGs*_*1–4*_) on the development of CM-related competence facets. Initially, whether the IG differed in these facets at baseline (*t*_*1*_) was assessed by computing analyses of variance (*ANOVAs*) and correlations between facets calculated. Development within each facet was analysed utilizing ANCOVAs estimating whether differences in the intervention explained variations in competence at t_2_ after controlling for the initial level. If significant differences were identified, group differences were examined by applying Tukey’s honestly significant test (*HSD*) test. Changes within each IG between t_1_ and t_2_ were analysed with paired *t*-tests and effects sized indicated by Cohens *d*. To ensure comparability across analyses, we repeated all analyses using the complete-case sample (*n* = 65), including only IPs with valid data across all outcome variables. Furthermore, we examined potential influences of the covariates: age, gender and intervention setting.

To explore whether improvements in knowledge and PID contribute independently to performance outcomes, we further extended the ANCOVA model by adding post-intervention scores of knowledge (*t*_*2*_) and PID (*t*_*2*_) as covariates. In doing so, we acknowledge that these covariates are not independent of the intervention assignment and therefore do not serve as control variables in the classical sense. Rather, their inclusion allows us to test whether knowledge and PID scores predict performance beyond the direct group-based intervention effects. This approach serves to explore potential mediator-like patterns without implying causal direction.

## Results

### Participant flow

A total of 89 PSTs were invited to participate in our study. Nine IPs were excluded prior to data analysis for various reasons (e.g., dropout, poor overall data quality). Leaving 80 IPs who provided valid data for either CM-related knowledge, PID, or performance, measured both before (*t*_*1*_) and after (*t*_*2*_) the interventions. However, the sample size in the analyses varied due to additional exclusions: for knowledge and PID, data from eight IPs were excluded because their completion times were shorter than the total vignette runtime, resulting in a final sample size of 72. For performance, data from seven IPs were excluded due to poor video quality, resulting in a final sample size of 73. As a result, the overlap of IPs with complete data on all three outcomes (knowledge, PID, and performance) was 65.

### Preliminary data analysis

Between the measurement points, the IPs in IG_1_ and IG_2_ reported teaching the subject of PE an average of 4.1 lessons (*SD* = 2.7), while IG_3_ and IG_4_ taught 3 lessons each, as part of the intervention design. To evaluate the quality of the video rating data, ICC values were calculated based on double ratings of 50% of the videos by five raters. The resulting ICC (*3, k*) were 0.88 (95% confidence interval [0.87, 0.90]) for the rater pair and 0.77 [0.72, 0.82], 0.79 [0.74, 0.83], 0.87 [0.84, 0.89] for the triplet, indicating good interrater reliability. One high outlier in the PID test at t_2_ was adjusted to the maximum value. Subsequent tests for the homogeneity of variance and normality of residuals were non-significant across groups, except for a significant Levene test result in the PID test at t_1_. However, no severe skewness or kurtosis was detected, confirming acceptable normality. At t_1_, the knowledge test (*EAP* = 0.69; *WLE* = 0.57) and the PID test (*EAP* = 0.73; *WLE* = 0.64) demonstrated acceptable reliability for group comparisons.

### Evaluation of the effectiveness of the interventions

ANOVAs of baseline measures showed no significant differences between IGs in knowledge (*F* (3, 68) = 2.29, *p* = 0.09), PID (*F* (3, 68) = 1.12, *p* = 0.35), or performance (*F* (3, 69) = 1.17, *p* = 0.33). The correlations between the facets were not statistically significant when tested across the full sample of IP at t_1_ (knowledge—PID: *r* = 0.16, *p* = 0.09; knowledge—performance: *r* = 0.15, *p* = 0.12; PID—performance: *r* = 0.051, *p* = 0.35).

To assess the effectiveness of the interventions on knowledge implemented in IGs_2–4_, we conducted an ANCOVA with knowledge at t_2_ as the outcome variable. The non-significant interaction term indicated that the effectiveness of the interventions did not statistically depend on IPs’ initial performance levels at t_1_, supporting the assumption of homogeneity of regression slopes. After adjusting for baseline knowledge at t_1_, no statistically significant group differences in knowledge at t_2_ were found (*F* (3, 64) = 1.41, *p* = 0.24). However, when the IG was redefined by separating IPs who received the knowledge intervention (*IG*_*2–4*_) from those who did not (*IG*_*1*_), a significant effect with a small effect size emerged (*F* (1, 68) = 4.16, *p* = 0.04, *η*^*2*^ = 0.04). Paired *t*-tests assessing within-group changes showed that CM-related knowledge in IG_1_ did not change significantly during the internship (*p* = 0.90, *d* = 0.03). In contrast, significant improvements were observed in IG_2_ (*p* = 0.02, *d* = 0.67), IG_3_ (*p* < 0.001, *d* = 1.12), and IG_4_ (*p* = 0.02, *d* = 0.46), indicating that intervention module 1 (see Sect. 4.3) effectively increased CM-related knowledge across these groups (see Table 1).

For PID, targeted in IG_3_ and IG_4_, ANCOVA results showed that, after adjusting for PID at t_1_, the PID results at t_2_ differed significantly for the different IGs, with a medium effect size (*F* (3, 64) = 8.62, *p* < 0.001, *η*^*2*^ = 0.09). Again, the interaction term was non-significant. Post-hoc analysis using Tukey’s HSD test corroborated the hypothesized effects, revealing significant differences in PID scores at t_2_ between IG_1_ and IG_3_, IG_1_ and IG_4_, as well as IG_2_ and IG_4,_ but not between IG_2_ and IG_3_. Paired *t*-tests indicated that PID increased significantly in IG_3_ (*p* = 0.004, *d* = 0.66) and IG_4_ (*p* = 0.03, *d* = 0.42) corresponding to the completion of intervention module 2 (see 4.3). In contrast, no significant changes were observed for IG_1_ (*p* = 0.91, *d* = 0.03) and IG_2_ (*p* = 0.75, *d* = 0.09).

Analysis of performance data using the ANCOVA showed that, after adjusting for performance at t_1_, performance ratings at t_2_ differed significantly for the different IGs with a medium effect size (*F* (3, 65) = 4.11, *p* = 0.01, *η*^*2*^ = 0.09). The non-significant interaction term again indicated that the effectiveness of the interventions did not statistically depend on IPs’ initial performance levels at t_1_. Tukey’s HSD post-hoc tests indicated significant differences between IG_4_ and IG_1_, IG_2_, and IG_3_, respectively. Paired *t*-tests revealed a significant improvement in performance only for IG_4_ (*p* = 0.004, *d* = 0.74), the only group that completed module 3 (see 4.3 and Table 2).

**Table 2:** Descriptive and inferential statistical results (within subjects) of IG_1–4_

Facet	IG_1_				IG_2_				IG_3_				IG_4_			
	*n*	*t*_*1*_ M (*SD*)	*t*_*2*_ M (*SD*)	*t*-test (*Cohens d*)	*n*	*t*_*1*_ M (*SD*)	*t*_*2*_ M (*SD*)	*t*-test (*Cohens d*)	*n*	*t*_*1*_ M (*SD*)	*t*_*2*_ M (*SD*)	*t*-test (*Cohens d*)	*n*	*t*_*1*_ M (*SD*)	*t*_*2*_ M (*SD*)	*t*-test (*Cohens d*)
CM-related knowledge	17	0.56 (*0.85*)	0.58 (*0.66*)	*p* = 0.90 (*0.03*)	13	0.25 (*0.78)*	0.78 (*0.80*)	*p* = 0.02* (*0.67*)	20	−0.05 (*0.74*)	0.64 (*0.89*)	*p* < 0.001*** (*1.12*)	22	0.49 (*0.86*)	0.88 (*0.88*)	*p* = 0.02* (*0.46*)
CM-related PID	17	−0.02 (*0.37*)	−0.01 (*0.60)*	*p* = 0.91 (*0.03*)	13	0.48 (*0.71*)	0.42 (0.*90*)	*p* = 0.38 (*0.09*)	20	0.25 (*0.86*)	0.74 (*0.98*)	*p* = 0.004** (*0.66*)	22	0.54 (*0.97*)	0.98 (*0.87*)	*p* = 0.03* (*0.42*)
CM-related performance	17	3.19 (*0.39*)	3.24, (*0.41)*	*p* = 0.40 (*0.21.*)	19	3.18 (*0.27*)	3.23 (*0.36*)	*p* = 0.16 (*0.23*)	20	3.35 (*0.32*)	3.33 (*0.39*)	*p* = 0.41 (*0.05*)	17	3.34 (*0.48*)	3.60 (*0.33*)	*p* = 0.004** (*0.74*)

*IG* Intervention group (see 4.3 and Fig. 2), *t*_*1*_ first measurement point (pre-test), *t*_*2*_ second measurement point (post-test)

*p < 0.05, **p < 0.01

Results based on the complete-case sample (*n* = 65) differed from the full sample in one outcome: the paired *t*-test assessing the improvement of CM-related knowledge in IG_4_ was not significant (*p* = 0.085, *d* = 0.35). Additional analyses of potential influence of the covariates age, gender and intervention setting revealed no significant effects.

After adjusting for baseline performance (*t*_*1*_) as well as knowledge (*t*_*2*_) and PID (*t*_*2*_), performance (*t*_*2*_) still differed significantly between groups (*F* (3, 55) = 4.26, *p* = 0.001, *η*^*2*^ = 0.11). However, neither knowledge (*t*_*2*_) nor PID (*t*_*2*_) significantly predicted performance, and their inclusion did not meaningfully improve the model’s explanatory power. The non-significant interaction terms confirmed that the relationships between covariates and performance did not differ significantly between groups, supporting the assumption of homogeneity of regression slopes.

## Discussion

### Answering the research question and hypotheses

This study investigated the extent to which the quality of the CM-related performances of PSTs (*DV*) can be improved through three developmental components: 1) improvement of CM-related knowledge, 2) enhancement of CM-related PID, and 3) implementation of the quality criteria of good CM in one’s own teaching practice (including instructor coaching, video-based performance feedback, self-reflection and training).

The results show that in IG_1_ the non-specific internship had no impact on the development of the (CM-related knowledge, CM-related PID, and) CM-related performances of PSTs, and therefore H_1_ is not rejected. This result aligns with existing findings, which demonstrates that general internships do not necessarily have a positive effect on the development of the competence or performance of PST, but rather that this requires specific structuring and focus (e.g., Bach 2013; Bastian et al. 2024; Baumgartner 2018b; Greve et al. 2020).

Moreover, the findings confirm that the quality of CM-related knowledge in IG_2_ significantly improved through targeted intervention (with a medium to large effect). However, in combination with the internship, this had no influence on the improvement of CM-related performance (and also no impact on the improvement of CM-related PID), which means that H_2_ must be rejected. This outcome contradicts the results of König and Pflanzl (2016) or Lenske et al. (2016). A possible explanation for the lack of effects observed in this study may lie in the type of knowledge tested (Lenske et al. 2016). When developing the test, care was taken to test only declarative knowledge and to exclude situated conditional-procedural knowledge. This decision was made to ensure that CM-related knowledge and PID could be empirically separated.

Regarding H_3_, the quality of CM-related knowledge and PID in IG_3_ significantly improved through targeted intervention (with a large and a medium to large effect), but that this had no effect on the improvement of CM-related performance in combination with the internship. Consequently, H_3_ is rejected. This result aligns with the diffuse and limited empirical evidence on the relationship between CM-related PID and performance. Unlike König and Kramer (2016), who uncovered significant relationships, Gold et al. (2021) and Junker et al. (2021) also failed to find a predictive effect of CM-related PID on CM-related performance, which aligns with our findings. A common feature of the studies, which showed no effects or correlations, is that they were conducted with PSTs or beginner teachers, whereas König and Kramer (2016) investigated in-service teachers. This possible explanation aligns with the conclusions of Bastian et al. (2023), who suggest that levels of PID increase significantly after entering the profession.

Ultimately, in IG_4_—analogous to IG_3_—the quality of CM-related knowledge and PID significantly improved through targeted intervention, each with moderate effects. In addition, the IPs in IG_4_ were able to significantly improve the quality of their CM-related performances through the intervention, with a moderate to large effect. A moderate to strong effect indicates that the intervention produces substantial changes (Cohen 1988). Accordingly, H_4_ is not rejected. This result corresponds with the findings of other studies (e.g., Baumgartner 2018b; Piwowar et al. 2016; Wilkinson et al. 2020). An additional analysis showed that post-intervention scores in knowledge and PID did not predict post-intervention performance when accounting for group membership and prior performance. This suggests that although both facets improved, they did not uniquely contribute to performance gains, nor act as mediators of the intervention effect. Given the exploratory nature of this analysis, we interpret these findings as indicative rather than causal.

Our findings demonstrate that the quality of CM-related performance can be improved by combining the following three development components: 1) improving CM-related knowledge, 2) enhancing CM-related PID, and 3) practising the implementation of the quality criteria of good CM in one’s own teaching practice (findings regarding *IG*_*4*_). For example, Wilkinson et al. (2020) demonstrate that improvements in CM-related performance are primarily driven by the effective components of coaching and performance feedback. In this context, training and self-reflection form the basis of the components. Compared to the other IGs, the intensity or the workload in IG_4_ was significantly higher, thereby possibly also resulting in a significant effect.

However, we were unable to demonstrate improvements in performance in IGs_1–3_. This suggests that the improvement of CM-related knowledge and CM-related PID does not per se increase PSTs CM-related performance in real-life situations. Given the quasi-experimental study design and the cumulative intervention design in IG_4_, which progressed from knowledge to PID to performance, an initial answer can also be given to the question raised by Blömeke et al. (2022) of whether the different competence facets should be acquired sequentially. Our findings suggest that this sequential approach is effective. Nevertheless, it cannot be discounted that an alternative intervention, focused on the implementation of the quality criteria of good CM in one’s own teaching practice to improve CM-related PID and knowledge, could also prove effective.

### Limitations of the study

This research investigated the development of the three CM-related competence facets (knowledge, PID, and performance) of PSTs at one university, at one school level, and in one field (PE). The results generated cannot automatically be generalized to other competence areas (e.g., feedback), to PSTs at other school levels, to those in different career phases (e.g., in-service teachers), or to other subjects.

In addition, it should be critically noted that the sampling procedures differed across the IGs. IG_1–2_ comprised two (out of 10) existing learning groups selected at random, whereas IG_3–4_ comprised PSTs who had voluntarily enrolled in a specialized PE internship. Despite these differing sampling approaches, it was found that the respective IGs did not differ at t_1_ with respect to the quality of the three facets.

Furthermore, it is not possible to differentiate the effects of the various intervention components in IG_4_ (e.g., instructor coaching, video-based performance feedback, training, and self-reflection) on performance improvement, as the intervention must be viewed *holistically*. Other influencing factors, such as affective-motivational aspects or self-efficacy beliefs, which were not controlled for in this investigation, may also have had an unknown impact on the results.

Additionally, the study is based on a relatively small sample size, which further limits the generalizability of the findings. Furthermore, due to the inclusion of only two measurement times, no conclusions can be drawn about the long-term development of the competence or performance of the PSTs, as a follow-up measurement was not conducted for reasons of economy. Lastly, it became evident that the quality of the three competence facets among the IPs was already relatively high at t_1_, meaning ceiling effects cannot be ruled out, and the intervention effect sizes may potentially have been limited by the high baseline values.

## Conclusion and future directions

This paper has shown that a cumulative acquisition of competencies along the competence continuum model developed by Blömeke et al. (2015) can improve the CM-related performance of PSTs. The different interventions demonstrated the expected intervention effects on the development of the targeted competence facets. Contrary to expectations, the findings show that PSTs with better CM-related knowledge and better PID do not provide a better CM-related performance, per se.

Future investigations could explore whether interventions regarding the explanatory construct of aspects of competency (e.g., involving a stronger integration of CM-related conditional-procedural knowledge) or PID that are more proximal to teaching in real-world settings (e.g., through an action intervention or through work with one’s own video vignettes [see Bastian et al. 2024]) would lead to significant effects on the facet of performance. A more detailed quasi-experimental study is needed to disentangle the specific developmental components within the cumulative intervention design IG_4_ that contributed to the observed large intervention effects. Further works should also examine whether less intensive (and realizable) intervention programmes in teacher education (e.g., by working with peer coaching through criteria-oriented and video-based feedback on one’s own teaching activities) lead to comparable effects. Studies that manipulate the various components (e.g., instructor feedback with video-based feedback versus instructor feedback without video) and examine their differential effects on performance development would be desirable. Moreover, the transferability of these results to other competence areas (e.g., feedback provided by PSTs) remains to be investigated, suggesting ample opportunities for future research. Moreover, the question arises whether correlations exist between the quality of teachers’ CM-related performance and student achievement.

Regarding the education of PE teachers—in effective university teaching and learning arrangements—a targeted combination of coursework and fieldwork (e.g., Baumgartner 2017; Brataas and Jenset 2023; Grossman et al. 2009) in which a connection to the PSTs’ own teaching practice is established proves to be effective. Based on this, diverse teaching and learning arrangements can be developed, in which the relevant components (e.g., coaching, video-based performance feedback, self-reflection, and training [Wilkinson et al. 2020]) are incorporated.

If increasing the performance of (PE) teachers is a goal of teacher education, the results indicate that teacher education (research) should not focus solely on the measurement and development of partial constructs (e.g., professional knowledge, PID), but rather more on the those of performance in real-life situations. Such a *practice-based turn in teacher education research*—whether related to competence or performance assessment, or intervention designs—would increase the ecological validity of the studies. The present work should be understood as a contribution to such a shift by supporting the design and implementation of practice- and evidence-based teacher education, in which PSTs learn to act professionally, as with in-service teachers.

## Footnotes

1 For a more in-depth clarification of the terminology used within the competence-oriented research paradigm, reference is made to the contribution by Baumgartner (2018a, 2022a).

2 In order not to exceed the scope of this paper, a more detailed discussion of CM in PE cannot be provided here. For a more in-depth examination of CM in PE, refer to Baumgartner et al. (2020).

3 The primary education in Switzerland corresponds to grades 1–6.
